# GATA3 regulates FLG and FLG2 expression in human primary keratinocytes

**DOI:** 10.1038/s41598-017-10252-x

**Published:** 2017-09-19

**Authors:** Jana Zeitvogel, Neele Jokmin, Samira Rieker, Ilona Klug, Christina Brandenberger, Thomas Werfel

**Affiliations:** 1 Division of Immunodermatology and Allergy Research, Department for Dermatology, Allergy and Venereology, Hannover Medical School, 30625 Hannover, Germany; 2 Institute of Functional and Applied Anatomy, Hannover Medical School, 30625 Hannover, Germany

## Abstract

GATA3 is a transcription factor with an important role in atopic diseases because of its role in the differentiation of Th2 lymphocytes. Moreover, GATA3 is expressed in keratinocytes and has a role in keratinocyte differentiation and the establishment of the epidermal barrier. In this study, we investigated the role of GATA3 in keratinocytes in the context of epidermal barrier integrity under inflammatory skin conditions. When analysing skin samples from atopic dermatitis and psoriasis patients or healthy controls, we detected decreased expression of GATA3 in the stratum spinosum and stratum granulosum of atopic dermatitis and psoriasis patients when compared to healthy controls. Our cell cultures experiments revealed that a downregulation in GATA3 by shRNA leads to a significant reduction of filaggrin mRNA under atopic dermatitis-like conditions in keratinocytes. Overexpression of GATA3 in keratinocytes reversed this effect and significantly upregulated filaggrin and, furthermore, filaggrin-2 mRNA expression. Our results demonstrate that GATA3 is involved in the regulation of filaggrin and filaggrin-2 expression during inflammatory conditions in the skin. Thus, GATA3 may be of special importance for the establishment and maintenance of an intact epidermal barrier, especially in atopic dermatitis.

## Introduction

The importance of the transcription factor GATA3 is well established for atopic diseases like allergic asthma bronchiale or atopic dermatitis^1,2^. Until now it has almost solely been associated with its function as a master transcription factor for Th2 differentiation and therefore as the responsible factor for the expression of the type 2 cytokines IL-4, IL-13 and IL-5 in T lymphocytes^3,4^. This function has made GATA3 a promising therapeutic target in Th2 cell based diseases like atopic dermatitis and allergic asthma. For the latter one, the therapeutic potential of GATA3 has recently been successfully demonstrated^5^.

Apart from its role in determining the fate of T cells, GATA3 is also highly expressed in the epidermis by keratinocytes^6–11^. In contrast to its functions in T cells, the role of GATA3 in human keratinocytes has been poorly investigated. In murine skin, GATA3 was shown to have important functions in the development and maintenance of an intact skin organ^12–14^. GATA3 has been shown to be essential for stem cell lineage determination and the establishment of the epidermal barrier and skin homeostasis during embryogenesis^12–14^. A mouse model with skin-specific GATA3 knock out revealed that without GATA3, the protective characteristics of the epidermal barrier are lost; mice with a lack of GATA3 in the skin die shortly after birth due to a dysplastic, non-functional skin with increased water loss^13^. The importance of GATA3 in the skin organ is not limited to embryogenesis; in the postnatal period, GATA3 holds important functions for the integrity of the epidermal compartment^12,13,15,16^. It is expressed in the nucleus of keratinocytes located in different epidermal layers^6–11^. Dependent on the study, GATA3 was reported to be expressed basal and suprabasal, in all layers, or in all layers except the granular and basal cell layers of the epidermis^6–11^. GATA3 was shown to be involved in the control of proliferation and differentiation of human and murine keratinocytes^6,7,17^. It affects the expression of several proteins including: human cytokeratin 1 and 10 as well as involucrin (IVL), loricrin (LOR), and kalikrein 1^6,15^. Recently, the expression of GATA3 in keratinocytes was linked to inflammatory skin diseases in humans; IL-4 was found to increase GATA3 levels in the epidermis whereas a reduced GATA3 expression was found in psoriatic lesions and under regenerative conditions^11^.

This study aims to examine the functions of GATA3 in the epidermal barrier in regards to chronic inflammatory skin diseases. We are in agreement with the finding of Racz (2011) that in psoriatic lesions the expression of GATA3 is reduced^11^. Moreover, by expanding the analysis to punch biopsies from atopic dermatitis lesions, we found a differing expression pattern of GATA3 in the epidermis of AD patients when compared to healthy controls. By modifying the expression of GATA3, we identified GATA3 as novel player in the regulation of filaggrin (FLG) and FLG2. Deregulated expression of FLG is well known to be one of the major pathogenetic factors in AD. Thus, the findings presented in this study point towards a crucial function for GATA3 in the integrity of the epidermal barrier in AD.

## Results

### GATA3 expression pattern is affected in lesional skin from patients suffering from atopic dermatitis

**Figure 1:**
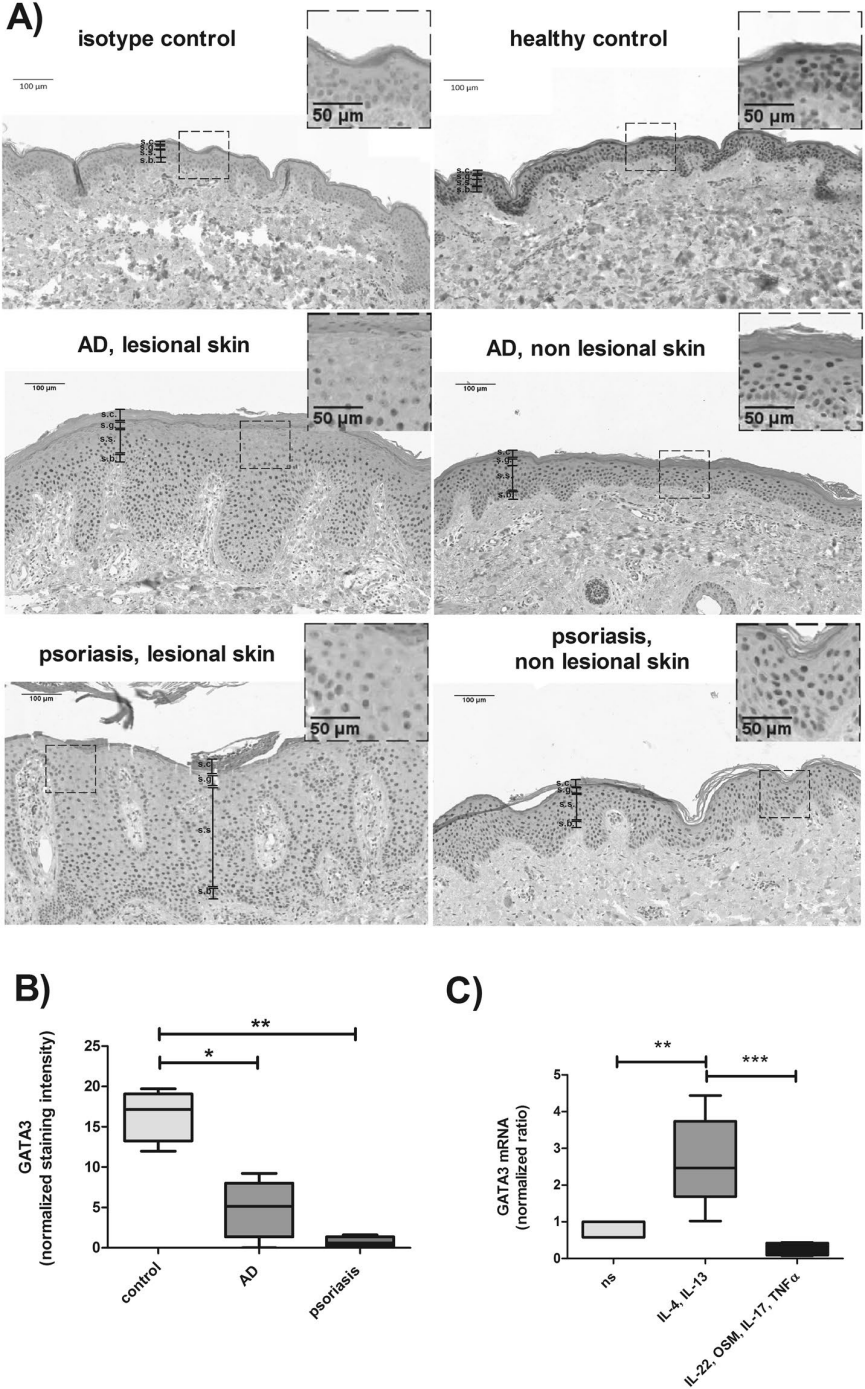
GATA3 expression profiles in skin from patients suffering from AD or psoriasis compared to healthy controls. (**A**) Skin biopsies from lesional and non-lesional skin as well as skin from healthy controls were stained for the expression of GATA3 by immunohistochemistry. A representative example of lesional and non-lesional skin (same donor) of AD and psoriasis as well as from a healthy control is depicted (psoriasis n = 4 (lesional and non-lesional skin), AD n = 3 (lesional and non-lesional skin) and 3 additional lesional AD samples were analysed). s.c. = stratum corneum, s.g. = stratum granulosum, s.s. = stratum spinosum, s.b. = stratum basale. (**B**) The intensity of the immunohistochemical staining of GATA3 in the three uppermost cell layers located in the stratum granulosum and stratum spinosum was quantified by using the software cellSense Dimension with Count & Measure Solution (Olympus). Cell layers located in the stratum corneum were not included in the quantification. The normalized staining intensity of GATA3 in lesional AD, lesional psoriasis and in healthy controls is depicted (normalized to the area of the analysed region). (**C**) Quantitative RealTime PCR was performed on primary keratinocytes cultivated with the indicated cytokines for 24 h (25 ng/ml were used of IL-4, IL-13, OSM and IL-17 and 10 ng/ml were used of IL-22 and TNFα), n = 5. Data are shown as box-and-whisker Tukey plots. P values depict statistical values estimated with the Holm-Sidak method *p < 0.05 and **p < 0.005.

Samples from lesional and non-lesional skin from patients suffering from atopic dermatitis or psoriasis as well as samples from healthy controls were analysed by immunohistochemistry staining for their expression patterns of GATA3 in the epidermis (Fig. 1A,B). In line with the publication of Racz *et al*.^11^, in healthy skin, GATA3 expression was detected in the nucleus of keratinocytes of all epidermal layers except the stratum corneum. Most prominent expression was found in the stratum spinosum. The samples of lesional skin from atopic dermatitis patients showed increased expression of GATA3 in the basal and suprabasal layers whereas the expression was reduced in the upper layers of stratum spinosum and stratum granulosum when compared to non-lesional skin and healthy controls (Fig. 1A). Furthermore, we confirmed the finding of Racz *et al*.^11^ that the expression of GATA3 is reduced in the epidermis of psoriatic lesions when compared to non-lesional skin and healthy controls (Fig. 1A). As depicted in Fig. 1B, the three upper most cell layers showed statistically significant differences in the mean values among the different groups (one-way Anova, p = 0,009). Multiple comparison post test revealed that the epidermis of lesional AD and psoriasis samples have a significant reduction in GATA3 expression in these cell layers when compared to healthy skin (AD vs. healthy p = 0,0107; psoriasis vs. healthy p = 0,00259 (both Holm-Sidak method)).

In cell culture experiments, we could mimic the GATA3 expression profiles seen in AD and psoriatic skin lesions by the use of disease specific stimuli (Fig. 1C). One-way repeated measures Anova revealed statistically significant differences in the mean values among the treatment groups (p = <0,001). Human primary keratinocytes stimulated with IL-4 and IL-13 (AD like conditions) showed a significant increase in GATA3 expression (p = 0,002, Holm-Sidak method) similar to the results obtained by immunohistochemical staining for the basal and suprabasal layers of lesional skin from AD patients depicted in Fig. 1A. In addition, the effect seen in psoriatic skin lesions could be reproduced in cell culture experiments by stimulation of keratinocytes with IL-22 (10 ng/ml), oncostatin M (OSM) (25 ng/ml), IL-17 (25 ng/ml), and TNFα (10 ng/ml) (psoriasis like conditions) which resulted in diminished GATA3 expression when compared to non-stimulated (ns) conditions (by trend) and significantly less GATA3 expression when compared to AD like conditions (p = 0,0003, Holm-Sidak method) (Fig. 1C).

### GATA3 silencing impacts genes located in the epidermal differentiation complex

**Figure 2:**
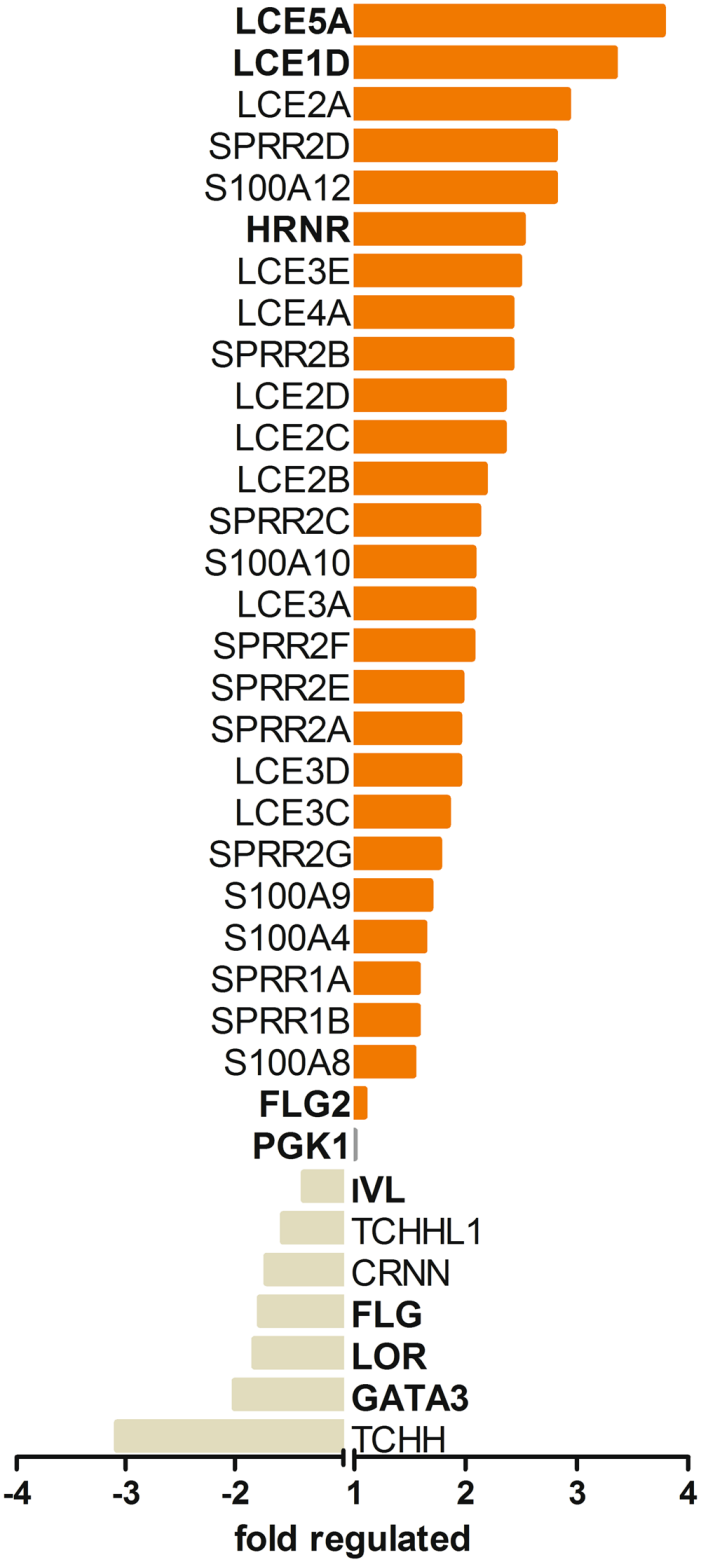
Microarray data from GATA3 silenced and control treated keratinocytes. Human primary keratinocytes were lentivirally transduced with either a vector encoding for a GATA3 targeting or a nonsense control shRNA. A human genome microarray (Agilent mRNA Microarrays (RCUT QuintQuad format), standard processing in single-color mode) was performed and attention was given to genes located in the human EDC (Ch1, 1q21). Genes that are located in the EDC and that were ≥1.5 fold regulated by GATA3 silencing or that were chosen for further analysis (written in bold letters) are depicted (late cornified envelope (LCE), small proline rich proteins (SPRR), trichohyalin (TCHH), cornulin (CRNN)). Furthermore, the fold regulation of GATA3 as well as that of phosphoglycerate kinase (PGK)1, which was used as housekeeping gene in this study, is displayed in the graph. The results reflect the mean of a sample of two pooled donors.

To further examine the role of GATA3 in the skin, we silenced GATA3 in human primary keratinocytes by lentiviral transduction of a vector that encodes for a GATA3 targeting shRNA. When compared to keratinocytes that were transduced with a nonsense control shRNA, GATA3 shRNA treatment resulted in a 50% average reduction in GATA3 mRNA levels (Fig. 2 and Supplementary Table S1). The mRNA expression of GATA3 in silenced keratinocytes and control cells was compared by microarray analysis. Special emphasis was given to genes that are important in the epidermal barrier. As depicted in Fig. 2, microarray data indicated that silencing of GATA3 impacts the expression of various genes located at the locus of the epidermal differentiation complex (EDC, human Chromosom 1, position 1q21; for a full list see supplementary Table S1). The expression of 20 genes were more than two fold regulated by GATA3 shRNA when compared to control cells (18 genes ≥2 fold upregulated, two genes ≥2 fold downregulated). The genes which showed more than two fold upreguation belong to the family of late cornified envelope proteins (LCE, 10 genes), S100 (2 genes), or small proline rich proteins (SPRR, 5 genes), whereas more than the half downregulated genes belong to the S100 fused gene family. The latter were trichohyalin (TCHH) and FLG. Hornerin (HRNR), another member of the S100 fused gene family, showed a 2.5 upregulation in expression, whereas FLG2 was not affected. IVL and LOR were only slightly influenced by GATA3 silencing (IVL was 1,37 fold and LOR 1,82 fold downregulated).

### GATA3 silencing impairs the integrity of the epidermal barrier

**Figure 3:**
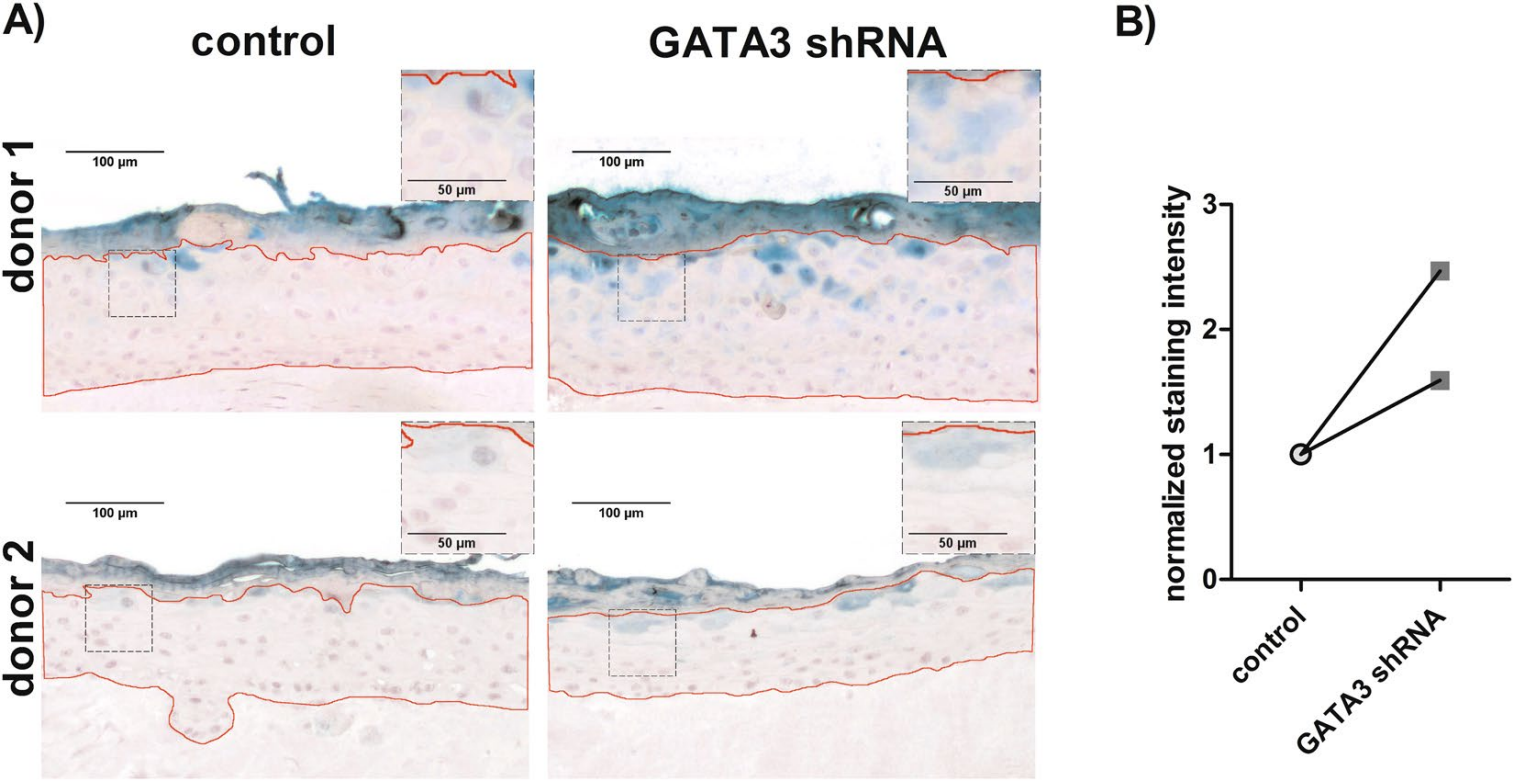
Penetration assay of GATA3 silenced and control cells. (**A**) 3D skin models were constructed from GATA3 silenced and control keratinocytes and a penetration assay was performed. The red-framed areas indicate the fields which were measured for the quantification depicted in 3B. The respective isotypes controls are depicted in Supplementary Fig. S1. (**B**) Immunohistochemical staining of the Lucifer Yellow treated 3D skin models was quantified; the normalized staining intensity of the Green solution is depicted. For quantification, the staining intensity of the epidermis with the exception of the stratum corneum (red-framed area delineated in 3 A) was measured and normalized to the area, n = 2.

To determine whether the effects of GATA3 silencing have a functional relevance for the epidermal barrier, we performed a dye penetration assay (Fig. 3). 3D skin equivalents were built up by using GATA3 silenced keratinocytes and keratinocytes transduced with a nonsense control. A drop of the dye was placed on the top of the epidermis and the skin equivalents were analysed by immunohistological staining for the presence and distribution of the dye in the epidermal compartment. As depicted in Fig. 3, GATA3 silencing resulted in a 2 fold increase in the penetration of the dye from the stratum corneum into the lower layers of the epidermis when compared to the control.

### GATA3 silencing affects involucrin and loricrin expression and significantly downregulates FLG expression under AD like conditions

**Figure 4:**
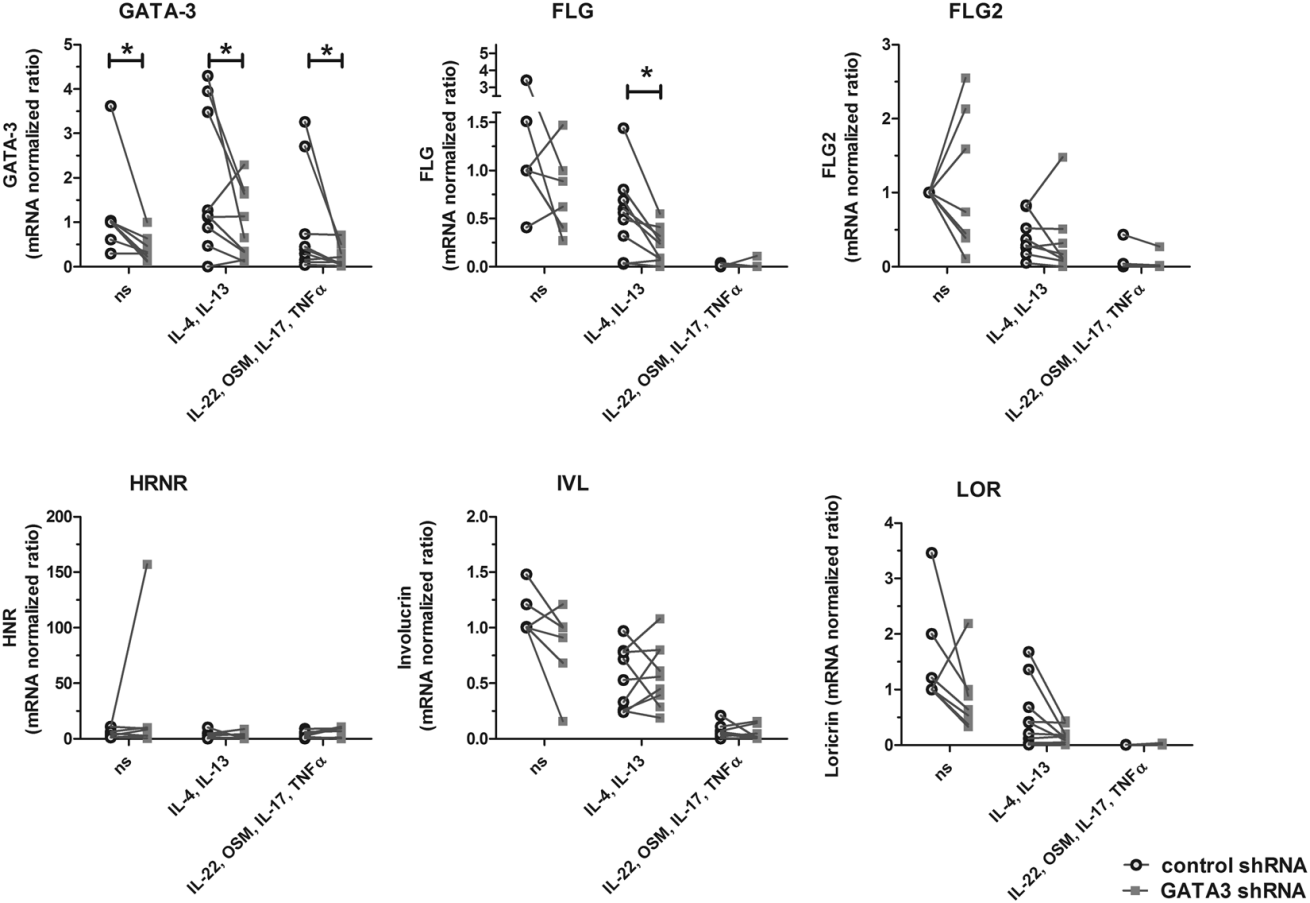
Influence of GATA3 silencing on the expression of genes encoding for major epidermal barrier proteins. GATA3 silenced and control treated keratinocytes were incubated with the indicated stimuli for 3 days (25 ng/ml were used for IL-4, IL-13, OSM and IL-17 and 10 ng/ml were used for IL-22 and TNFα). Subsequently, qRT-PCR was performed and the expression of GATA3, LOR, IVL, FLG, FLG2, and HRNR were analysed. Relative mRNA expression levels normalized to PGK1 are shown. n = 7–8 independent experiments with keratinocytes from different donors. P values depict statistical values estimated with t-test (paired-t-test or Wilcoxon signed rank test). *p < 0.05.

We decided to further investigate the impact of GATA3 on the expression of FLG, FLG2, HRNR, IVL, and LOR (Fig. 4). We decided to focus on these genes because of their known importance in the epidermal barrier, especially in atopic dermatitis and psoriasis. They also represent differentiation markers of different epidermal layers and allow for an assessment of the involvement of GATA3 in the different differentiation steps. Moreover, by reasons of the high influence of the GATA3 silencing on LCE1E and LCE5A expression indicated by the microarray, LCE1E and LCE5A were chosen for further analysis (Supplementary Fig. S2). To validate the data obtained by microarray, we performed qRT-PCR and included AD like conditions (IL-4 and IL-13 treatment) as well as psoriasis like conditions (IL-22, OSM, IL-17, and TNFα treatment) in our experimental setup. The expression of the target genes in GATA3 silenced cells (GATA3 shRNA) was compared to control cells transduced with a nonsense shRNA (control shRNA). As depicted in Fig. 4, in all three tested conditions (ns, AD like conditions, psoriasis like conditions) GATA3 silencing resulted in significantly reduced GATA3 expression when compared to the control cells (GATA3 shRNA vs control: ns p = 0,0156; IL-4, IL-13 p = 0,0391; IL-22, OSM, IL-17, TNFα p = 0,0391 (all Wilcoxon signed rank test)). We confirmed the tendencies seen in the microarray for FLG, LOR, and IVL mRNA expression. All three genes showed reduced expression in GATA3 silenced cells when compared to the control cells by trend. Interestingly, under AD like conditions (IL-4, IL-13) FLG expression was significantly reduced in the GATA3 silenced cells when compared to the control cells (p = 0,0289, paired t-test). In contrast, in case of IVL, the tendency of a reduced involucrin expression in GATA3 silenced cells when compared to control cells as seen under ns condition was abrogated by IL-4 and IL-13 stimulation. No tendency for divergent gene expression of FLG, FLG2, IVL, LOR or HRNR in GATA3 silenced cells when compared to control cells was visible under psoriasis-like conditions. Possible effects of GATA3 silencing might be superimposed by the treatment of the cells with IL-22, OSM, IL-17, and TNFα. In line with the microarray data, FLG2 expression was not significantly altered by GATA3 silencing in the qRT-PCR analysis when compared to the control. We could not validate the findings of the microarray for LCE1E, LCE5A (see Supplementary Fig. S2) and HRNR (Fig. 4) – although there were differences between the control and the GATA3 silenced cells, no clear tendencies were detectable.

### GATA3 overexpression significantly upregulates FLG and FLG2 expression

**Figure 5:**
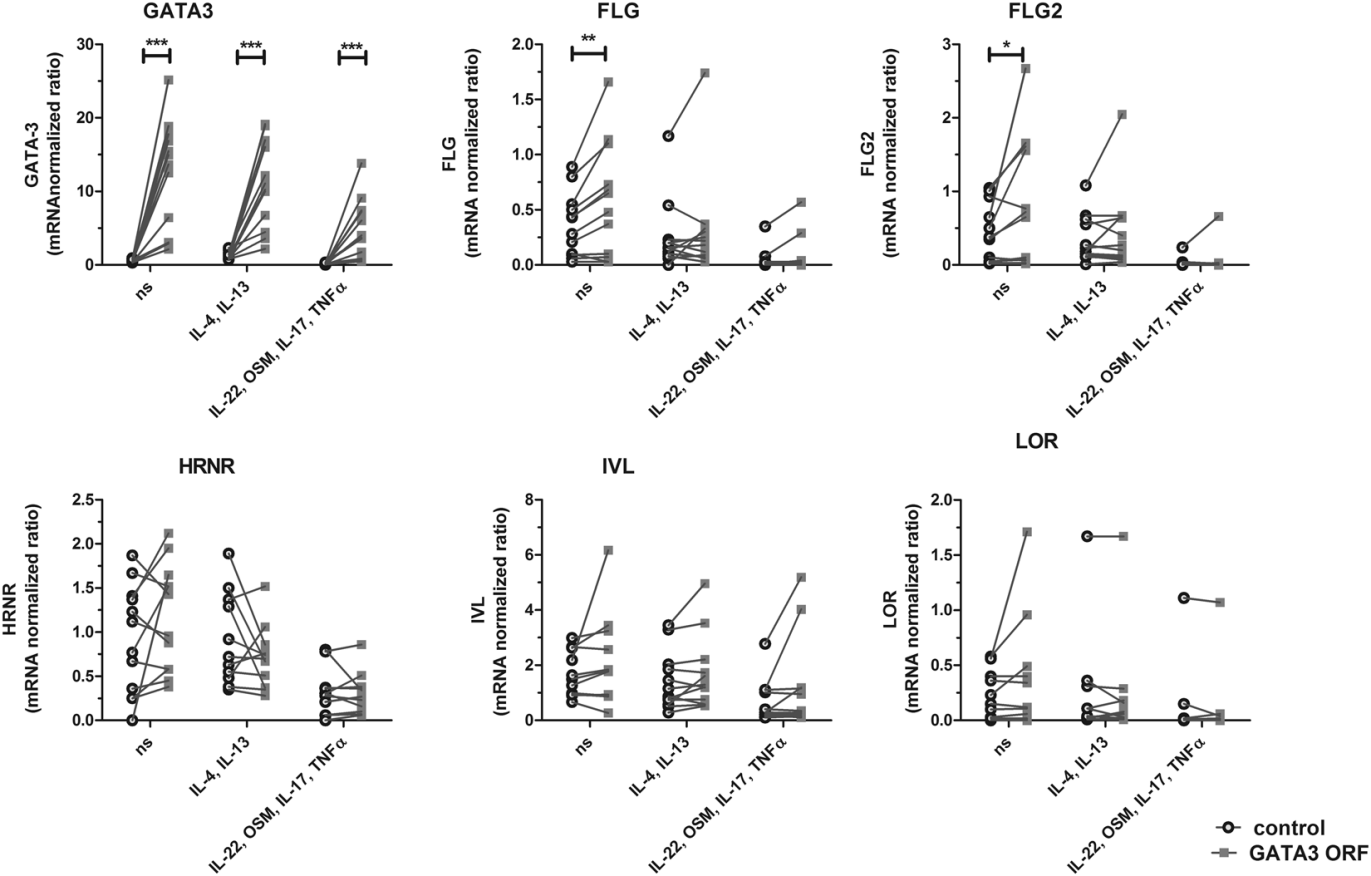
Influence of GATA3 overexpression on the expression of genes encoding for major epidermal barrier proteins. Human primary keratinocytes were transfected with a plasmid encoding either for the ORF of human GATA3 or firefly luciferase as control, respectively. 48 h post transfection cells were stimulated with the indicated stimuli for 24 h (25 ng/ml were used of IL-4, IL-13, OSM, and IL-17 and 10 ng/ml were used of IL-22 and TNFα). Subsequently, qRT-PCR was performed and the expression of GATA3, LOR, IVL, FLG, FLG2, and HRNR were analysed. Relative mRNA expression levels normalized to PGK1 are shown. n = 12 independent experiments with keratinocytes from different donors. P values depict statistical values estimated with t-test (paired-t-test or Wilcoxon signed rank test). *p < 0.05, **p < 0.005 and ***p < 0.0005.

GATA3 was overexpressed in human primary keratinocytes to further prove the role of GATA3 in the expression of FLG, FLG2, HRNR, IVL, and LOR (Fig. 5). Primary human keratinocytes were transfected with a plasmid encoding the open reading frame (ORF) of GATA3 or a control. We confirmed the overexpression of GATA3 by FACS analysis (data not shown) and qRT-PCR, which resulted in a significant increase in GATA3 expression under all investigated conditions (GATA3 ORF compared to control ORF: ns p = 0,0001(paired t-test); IL4, IL-13 p = 0,0005 (Wilcoxon signed rank test); IL-22, OSM, IL-17, TNFα p = 0,0017 (paired t-test); Fig. 5). Subsequently, the mRNA expression of FLG, FLG2, HRNR, IVL, and LOR was analysed and cells overexpressing GATA3 were compared to control cells (Fig. 5). We found a significant upregulation in FLG and FLG2 by GATA3 overexpression when compared to cells transfected with a control plasmid under ns conditions (FLG p = 0,0049 and FLG2 p = 0,0273 (both Wilcoxon signed rank test)). This effect could not be sustained by stimulation with IL-4 and IL-13 or IL-22, OSM, IL-17, and TNFα. No significant effects were found for LOR, IVL and HRNR expression when comparing GATA3 ORF cells to cells transfected with a control ORF (Fig. 5).

### Decreased FLG and FLG2 protein expression in 3D skin equivalents comprised GATA3 silenced keratinocytes

**Figure 6:**
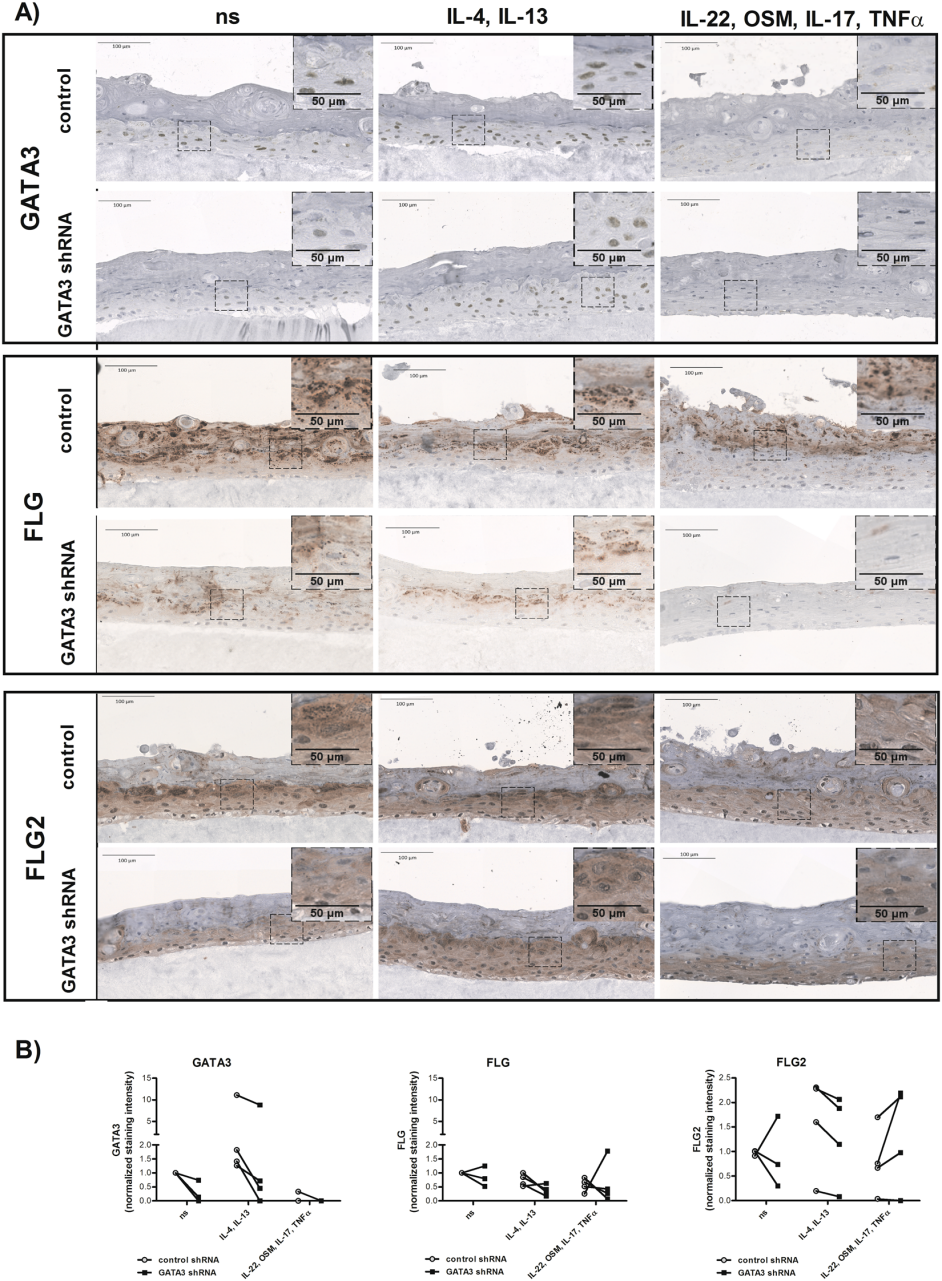
Immunohistochemical analyses of the effect of GATA3 silencing on the protein expression of FLG and FLG2. (**A**) 3D skin equivalents were constructed from GATA3 silenced or control treated cells. The 3D models were incubated for 3 days in the presences of the indicated cytokines (25 ng/ml were used of IL-4, IL-13, OSM, and IL-17 and 10 ng/ml were used of IL-22 and TNFα). Afterwards immunohistological staining was performed to detect GATA3, FLG, and FLG2, respectively. Representative examples of 3–4 independent experiments with different donors are shown. (**B**) Immunohistochemical stainings of GATA3, FLG and FLG2 were quantified by using the software cellSense Dimension with Count & Measure Solution (Olympus). The normalized staining intensity of AEC and DAB, respectively, is depicted (normalized to the area of the section).

GATA3 shRNA transduced keratinocytes and control cells were used to generate 3D skin equivalents (Fig. 6). The 3D skin equivalents were incubated, up on day four for the following three days under AD like conditions (IL-4 and IL-13), psoriasis like conditions (IL-22, OSM, IL-17, and TNFα) or left untreated. After this period, the skin models were fixed in formalin and used for immunohistological staining to analyse the expression of GATA3, FLG and FLG2 at protein level. As depicted in Fig. 6A and B, we observed less GATA3 protein in 3D skin equivalents made with GATA3 silenced keratinocytes than in the skin models comprised of control cells under ns and psoriasis like conditions by trend. 3D skin equivalents made of GATA3 shRNA transduced cells showed the tendency to express less FLG when compared to the respective control 3D skin equivalent under all investigated conditions (ns, AD-like (IL-4 and IL-13), and psoriasis-like (IL-22, OSM, IL-17, and TNFα) conditions). In the case of FLG2, silencing of GATA3 led to decreased FLG2 levels under AD like conditions when compared to the control models. In contrast, 3D skin equivalents made of GATA3 silenced keratinocytes which were cultivated under psoriasis like conditions showed increased FLG2 protein levels when compared to the correlated controls (controls shRNA) by trend.

## Discussion

Here we show for the first time that GATA3 is involved in the regulation of FLG and FLG2 in human primary keratinocytes.

Recent publications have pointed to an important role of GATA3 in the establishment and maintenance of an intact epidermal barrier^6,13,15^. GATA3 was assigned a particular function in the keratinocyte transition state from proliferating to differentiating^6^. This function of GATA3 was linked to the p63/IKKα signalling pathway^7^; in mice p63, a well-known regulator of epidermal development and keratinocyte differentiation, was shown to activate GATA3 gene expression. Subsequently, GATA3 binds to the IKKα promotor and activates its expression which results in the differentiation of keratinocytes^7,17^. Amongst others IKKα was shown to be necessary for the expression of FLG and LOR^18–20^. In line with these observations, GATA3 knock out mice show a similar skin phenotype to that of p63 knock out mice. Both mice are characterized by a loss of functionality in the epidermal barrier; the animals die shortly after birth owing to dehydration which was due to a high trans epidermal water loss^13,21,22^. Of note, our human microarray data showed no reduction in IKKα levels in GATA3 silenced keratinocytes when compared to control cells (data not shown). This points either to other regulatory elements involved in the activation of IKKα expression, which are able to replace GATA3, or to species dependent differences in the regulation of IKKα. In conclusion the silencing of GATA3 impairs the differentiation of human keratinocytes which is seen in the reduced expression of FLG, IVL, and LOR.

FLG and FLG2 were significantly upregulated in human keratinocytes that overexpressed GATA3. This points towards a direct function of GATA3 in the activation of FLG and FLG2 expression. Of note, in contrast to FLG, FLG2 expression was not positively altered in the GATA3 silenced cells which might be due to other factors that are able to replace GATA3. Therefore, we conclude that in our cell culture conditions, GATA3 is sufficient but not necessary to activate FLG2 expression whereas GATA3 is necessary and sufficient for the activation of the FLG promoter.

Of importance, we found that GATA3 expression levels are altered in lesional skin of AD patients. This strengthens the impact of our observation that GATA3 regulates FLG expression in the pathogenesis of AD. Defects in skin barrier properties and in particularly dysregulated FLG expression are considered to be a hallmark of AD^23,24^. A reduced GATA3 expression in skin of AD patients may boost the permeability of the skin. A potential increase in the uptake of pathogens or substances with an irritating or allergic potential is probable which could promote the development of chronic skin inflammation. Although we observed an upregulation of GATA3 in the stratum basale and in the lower layers of the stratum spinosum in lesional AD skin, the expression in the upper layers of the stratum spinosum and in the stratum granulosum was still lower than seen in healthy controls. Furthermore, the effect of GATA3 overexpression was not powerful enough to abrogate the suppressing effects of IL-4 and IL-13 on the FLG mRNA expression. This may explain why the expression of FLG is not increased in epidermal layers with enhanced GATA3 expression in lesional AD skin. A strong inhibitory effect of IL-4 and IL-13 on FLG expression was already reported before by Howell *et al*.^25^ and has been reproduced by many groups. The reduced GATA3 expression in the upper layers of the stratum spinosum and stratum granulosum described here may further contribute to the reduced FLG expression in lesional skin of AD patients.

In contrast to FLG, overexpression of GATA3 did not upregulate IVL and LOR in a similar manner. Therefore, we assume that GATA3 is necessary for an optimal but not sufficient for gene transcription of IVL and LOR. Of note, in mice GATA3 was found to be directly involved in the regulation of LOR expression by two ways: First, by binding to a positive regulatory element in the proximal promoter region of the gene, and secondly, by interacting with the transcription factors SP1 and c-Fos to enhance the activation of the loricrin promoter^16^. However, GATA3 is not necessary for LOR expression; a combination of the transcription factors SP1 and c-Fos were shown to be sufficient to activate the loricrin promoter in mice^26^. A similar but even more complex interplay from different transcription factors was reported to be responsible in human regulation of LOR gene expression; SP1, c-Jun, an unidentified regulator which binds to a CRE-like site, and the co-activator p300/CREB-binding protein up regulates, whereas SP3, CREB-1/CREAMα/ATF-1, Jun B, and an AP2-like protein suppresses loricrin promoter activity^27^. Most of these transcription factors are considered to be ubiquitously transcribed, but Mehic *et al*.^28^ reported a differentiation dependent expression of the AP1 proteins in human keratinocytes. IVL gene regulation was also reported to be activated by AP1 and SP1 in addition to CCATT/enhancer-binding protein^29–33^. Thus, a missing AP1 factor in GATA3 overexpressed keratinocytes may explain the lack of efficiently enhanced LOR and IVL gene transcription.

Taken together, we have identified GATA3 as an important factor in the regulation of FLG and FLG2 expression. This should be considered in the therapy of AD, particularly in regards to corticosteroids known to suppress GATA3 function and expression^34^.

## Methods

### Cytokines and reagents

All cytokines were used as purified recombinant human preparations. IL-4 and IL-13 were purchased from R&D Systems (Wiesbaden, Germany), IL-17, IL-22, and OSM were ordered from Peprotech (Hamburg, Germany). TNFα were purchased from BioLegend (San Diego, USA). The Rho kinase inhibitor Y27632 was purchased from Abcam (Cambridge, UK).

### Human samples

Biopsy specimens (3–4 mm punch specimens) were taken from patients with atopic dermatitis or psoriasis. Control samples were received from plastic surgeries (abdominoplasty, reduction mammoplasty) from skin healthy donors. The study was approved by the Medical Ethics Committee of the Hannover Medical School and was conducted according to the Declaration of Helsinki Principles. All patients provided written informed consent.

### Transduction and transfection

Human primary keratinocytes that were prepared from foreskin (as described before^35^) were used for transduction when reaching 40% confluency. Cells were cultured and transduced in keratinocyte medium (Keratinocyte Growth Medium 2 Kit; PromoCell, Heidelberg, Germany). For transduction 3 µg/ml Polybrene (Merck/Milliipore, Darmstadt, Germany) was added to the media and cells were transduced overnight by adding the viral particles at a MOI of 16. Viral particles were purchased from GE Healthcare (Little Chalfont, UK) (Smartvector 2.0 Non-Targeting control particles or SMARTchoice Lentiviral shRNA for GATA3). Selection was performed in keratinocyte medium containing 1 µg/ml puromycin (ThermoFisher Scientific, Darmstadt, Germany).

Overexpression of GATA3 was performed using the vector EX-F0716-M61 and EX-hLUC-M61 as control, respectively (both from GeneCopoeia, Rockville, USA). Keratinocytes were used for transfection when their confluency was around 80%. For transfection FugeneHD (Promega, Mannheim, Germany) was used. 48 hours post transfection cells were stimulated for 24 hours with IL-4 and IL-13 (both 25 ng/ml, AD like conditions) or IL-22 (10 ng/ml), OSM (25 ng/ml), IL-17 (25 ng/ml), and TNFα (10 ng/ml) (psoriasis like conditions), or left untreated. After this stimulation period, cells were lysed and analysed by qRT-PCR.

### Cell culture and 3D cultures of transduced keratinocytes

Transduced cells were cultured in keratinocyte medium (Keratinocyte Growth Medium 2 Kit; PromoCell) which was supplemented with 10 µM Y27632 to increase the capacity of the cells to proliferate^36,37^. When cells reached 80% confluency, they were passaged or used for downstream assays. Cells that were plated for experiments were incubated during the further procedure without Y27632. After reaching confluency they were stimulated with either IL-4 and IL-13 (both 25 ng/ml, AD like conditions) or IL-22 (10 ng/ml), OSM (25 ng/ml), IL-17 (25 ng/ml), and TNFα (10 ng/ml) (psoriasis like conditions) for an additional three days. After this stimulation period cells were lysed and analysed by qRT-PCR.

Transduced cells were further used for 3D skin equivalents. 3D skin equivalents were built as described before^38^. The skin models were stimulated on day four with IL-4 and IL-13 (both 25 ng/ml) or IL-22 (10 ng/ml), OSM (25 ng/ml), IL-17 (25 ng/ml), and TNFα (10 ng/ml), or left untreated for further three days. After this period, 3D skin equivalents were fixed in formalin, embedded in paraffin and used for immunohistochemical staining.

### Penetration assay

For the penetration assay, non-stimulated 3D skin equivalents were used on day 7. A drop (20 µl) of the dye Lucifer Yellow (Sigma-Aldrich, St. Louis, USA) was added to the epidermis for two hours. Subsequently, the dye was carefully removed with a cotton bud and the 3D models were fixed in formalin and embedded in paraffin. As the transduced cells also express GFP as selection marker, the dye had to be stained in a different colour to visualize possible differences in the penetration efficiency. Therefore, immunohistochemical staining with an antibody against Lucifer yellow was performed.

### Immunohistological staining

For the detection of GATA3, a monoclonal rabbit antibody (Cell Signalling Technologies) was used. FLG was stained with a monoclonal mouse antibody purchased from Abcam and for FLG2detection, a polyclonal rabbit antibody (Sigma-Aldrich) was used. For all three targets the Envision+ kits from Dako (Hamburg, Germany) were used (FLG: HRP.Mouse (AEC+); GATA3: HRP.Rabbit (DAB+); FLG2: HRP.Rabbit AEC+)). The detection of Lucifer Yellow was performed with a polyclonal rabbit antibody (bought from ThermoScientific) in combination with HRP-Green Solution Set (42 life sciences GmbH & Co. KG, Bremerhaven, Germany). In all experiments the corresponding isotype control was added as a control. For the staining of FLG, GATA3, and Lucifer Yellow the “Target Retrieval Solution, ph9” and for FLG2 the “Target Retrieval Solution, ph6” (both Dako) was used. An Axio Scan.Z1 (Zeiss, Jena, Germany) was used for digitalization of the immunohistological specimens depicted in this study. Quantification of the staining intensity was done by using the software cellSense Dimension with Count & Measure Solution (Olympus Deutschland GmbH, Hamburg, Germany).

### mRNA isolation and qRT-PCR

RNA was isolated using the “High Pure RNA Isolation Kit” (Roche Molecular Biochemicals, Mannheim, Germany) or the “innuPREP Micro RNA Kit” (AnalitikJena, Jena, Germany). Reverse transcription was performed using the “QuantiTect Reverse Transcription Kit” (Qiagen, Hilden, Germany). The qRT-PCR was performed either on a LightCycler480 (Roche Molecular Biochemicals) or on a RotorGene (Qiagen) using the “LightCycler® 480 SYBR Green I Master” (Roche Molecular Biochemicals). The primers for FLG, FLG2, IVL, LOR, HRNR and PGK1 were bought from Qiagen (“QuantiTect Primers”). For quantitative analysis, targets were quantified using relative quantification (ΔΔCT method) (software either from Roche Molecular Biochemicals or Qiagen, dependent on the used machine for qRT-PCR).

### Microarray analysis

A human genome microarray (Agilent mRNA Microarrays, Agilent Technologies Deutschland GmbH, Waldbronn, Germany) was performed in RCUT QuintQuad format. The microarray was run on an Agilent Microarray Scanner G2565CA (Agilent Technologies Deutschland GmbH) under standard processing conditions and in single-color mode.

### Statistical analysis

For comparison of more than two groups, one-way Anova and multiple comparisons post-test according to the Holm-Sidak method was performed by using the software SigmaStat for Windows 3.5 (Systat Software, Inc., Erkrath, Germany). For the comparison of two groups, data were either analysed using paired t-test or Wilcoxon Signed Rank Test according to the distribution of the data. These statistical analyses were performed with the software GraphPad Prism version 5.0a. Asterisks in the figures indicate the p values: *p < 0.05, **p < 0.005 and ***p < 0.0005.

### Data availability

The datasets generated during and/or analysed during the current study are available from the corresponding author on reasonable request.

## Electronic supplementary material

Supplementary Information
