# Supplementary material for: GATA3 regulates FLG and FLG2 expression in human primary keratinocytes
Source: Sci Rep. 2017 Sep 19;7:11847. doi: 10.1038/s41598-017-10252-x (PMC5605628; doi:10.1038/s41598-017-10252-x)
Supplement: Supplementary file 1 — Supplementary Information [file 41598_2017_10252_MOESM1_ESM.pdf]

SUPPLEMENTAL DATA

Manuscript:

GATA3 regulates FLG and FLG2 expression in human primary keratinocytes

Jana Zeitvogel[1*], Neele Jokmin[1], Samira Rieker[1], Ilona Klug[1], Christina Brandenberger [2], and Thomas Werfel[1]

[1]Division of Immunodermatology and Allergy Research, Department for Dermatology, Allergy and Venereology, Hannover Medical School, 30625 Hannover, Germany

[2]Institute of Functional and Applied Anatomy, Centre of Anatomy and Cell Biology, Hannover Medical School, 30625 Hannover, Germany

***Corresponding Author:**

Jana Zeitvogel

Division of Immunodermatology and Allergy Research, Department for Dermatology, Allergy and Venereology, Hannover Medical School

Carl-Neuberg-Str. 1

D-30625 Hannover, Germany

Email: Zeitvogel.Jana@MH-Hannover.de

Gene Symbol	Gene Name	Systematic Name	Ratio (Gata3 shRNA / control shRNA)
Epidermal differentiation complex:			
Late cornified envelop family:			
LCE1A	Late cornified envelope 1A	NM_178348	1,16
LCE1B	Late cornified envelope 1B	NM_178349	1,10
LCE1C	Late cornified envelope 1C	NM_178351	1,57
LCE1D	Late cornified envelope 1D	NM_178352	3,34
LCE1E	Late cornified envelope 1E	NM_178353	1,46
LCE2A	Late cornified envelope 2A	NM_178428	2,92
LCE2B	Late cornified envelope 2B	NM_014357	2,17
LCE2C	Late cornified envelope 2C	NM_178429	2,34
LCE2D	Late cornified envelope 2D	NM_178430	2,34
LCE3A	Late cornified envelope 3A	NM_178431	2,07
LCE3B	Late cornified envelope 3B	NM_178433	1,44
LCE3C	Late cornified envelope 3C	NM_178434	1,84
LCE3D	Late cornified envelope 3D	NM_032563	1,94
LCE3E	Late cornified envelope 3E	NM_178435	2,48
LCE4A	Late cornified envelope 4A	NM_178356	2,41
LCE5A	Late cornified envelope 5A	NM_178438	3,77
LCE6A	Late cornified envelope 6A	NM_001128600	1,21
S100 calcium binding protein family:			
S100A1	S100 calcium binding protein A1	NM_006271	1,02
S100A2	S100 calcium binding protein A2	NM_005978	1,21
S100A3	S100 calcium binding protein A3	NM_002960	-1,01
S100A4	S100 calcium binding protein A4	NM_002961	1,63
S100A5	S100 calcium binding protein A5	NM_002962	-1,33
S100A6	S100 calcium binding protein A6	NM_014624	1,27
S100A7	S100 calcium binding protein A7	NM_002963	-1,47
S100A7A	S100 calcium binding protein A7A	NM_176823	-1,11
S100A7L2	S100 calcium binding protein A7-like 2	NM_001045479	1,00
S100A8	S100 calcium binding protein A8	NM_002964	1,53
S100A9	S100 calcium binding protein A9	NM_002965	1,68
S100A10	S100 calcium binding protein A10	NM_002966	2,07
S100A11	S100 calcium binding protein A11	NM_005620	1,42
S100A12	S100 calcium binding protein A12	NM_005621	2,80
S100A13	S100 calcium binding protein A13	NM_001024210	1,35
S100A14	S100 calcium binding protein A14	NM_020672	1,18
S100A16	S100 calcium binding protein A16	NM_080388	1,15

Gene Symbol	Gene Name	Systematic Name	Ratio (Gata3 shRNA / control shRNA)
S100 fused-type protein family			
TCHH	Trichohyalin	NM_007113	-3,08
TCHHL1	Trichohyalin-like 1	NM_001008536	-1,56
CRNN	Cornulin	NM_016190	-1,71
RPTN	Repetin	NM_001122965	1,00
HRNR	Hornerin	NM_001009931	2,51
FLG	Filaggrin	NM_002016	-1,77
FLG2	Filaggrin family member 2	NM_001014342	1,09
Small proline-rich proteins			
SPRR1A	Small proline-rich protein 1A, transcript variant 2	NM_005987	1,57
SPRR1B	Small proline-rich protein 1B	NM_003125	1,57
SPRR2A	Small proline-rich protein 2A	NM_005988	1,94
SPRR2B	Small proline-rich protein 2B	NM_001017418	2,41
SPRR2C	Small proline-rich protein 2C (pseudogene) , non-coding RNA	NR_003062	2,11
SPRR2D	Small proline-rich protein 2D	NM_006945	2,80
SPRR2E	Small proline-rich protein 2E	NM_001024209	1,96
SPRR2F	Small proline-rich protein 2F	NM_001014450	2,06
SPRR2G	Small proline-rich protein 2G	NM_001014291	1,76
SPRR3	Small proline-rich protein 3, transcript variant 1	NM_005416	1,44
SPRR4	Small proline-rich protein 4	NM_173080	1,48
Others			
IVL	Involucrin	NM_005547	-1,37
LOR	Loricrin	NM_000427	-1,82
Silenced Gene:			
GATA3	GATA binding protein 3	NM_001002295	-2,00
Housekeeping Gene used in this study:			
PGK1	Phosphoglycerate kinase 1	NM_000291	1,00

Supplementary Table S1: Microarray data from GATA3 silenced and control treated keratinocytes. Human primary keratinocytes were lentiviral transduced with either a vector encoding for a GATA3 targeting or a nonsense control shRNA. A human genome microarray (Agilent mRNA Microarrays (RCUT QuintQuad format), standard processing in single-color mode) was performed and attention was turned to genes located in the human epidermal differentiation complex (Ch1, 1q21) as depicted. Genes that were ≥ 1.5 fold upregulated are highlighted in orange and those which were ≥ 1.5 fold downregulated are highlighted in green. Genes which gene symbols are written with underlined letters were chosen for further analysis in this study. Furthermore the fold regulation of GATA3 as well as of the housekeeping gene PGK1 used in this study is displayed in the table. The results reflect the mean of a sample of two pooled donors.

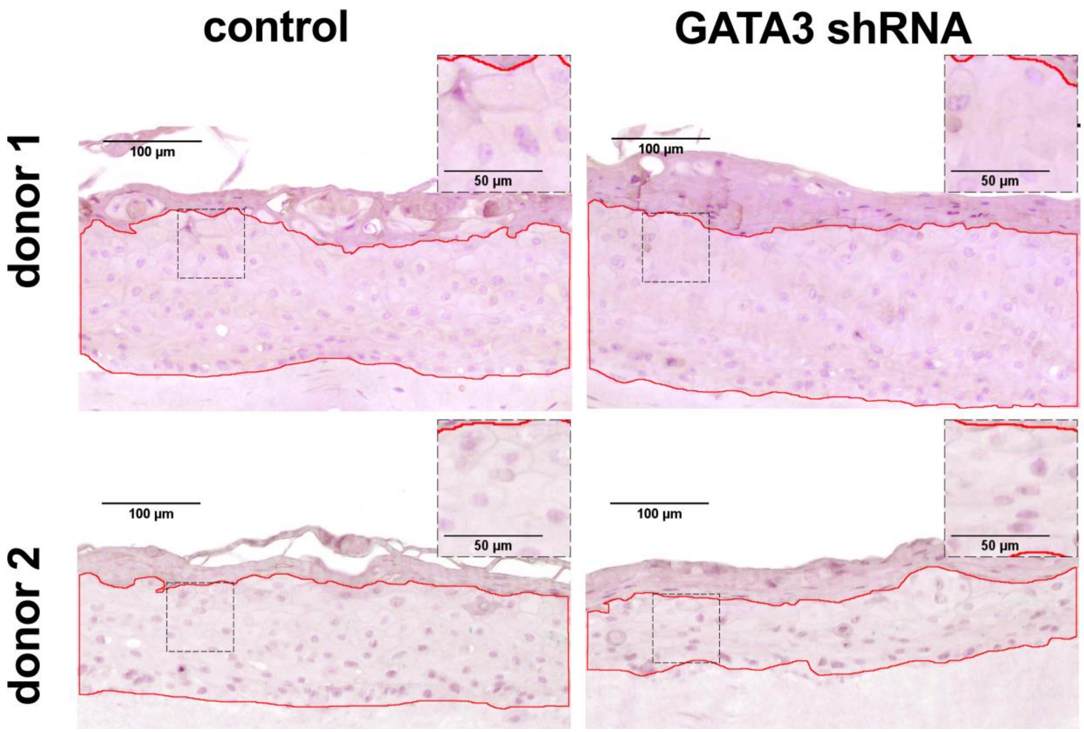

Supplementary Figure S1: Penetration assay of GATA3 silenced and control cells. 3D skin models were constructed from GATA3 silenced and control keratinocytes and a penetration assay was performed. The respective isotypes controls to Fig. 3 are depicted.

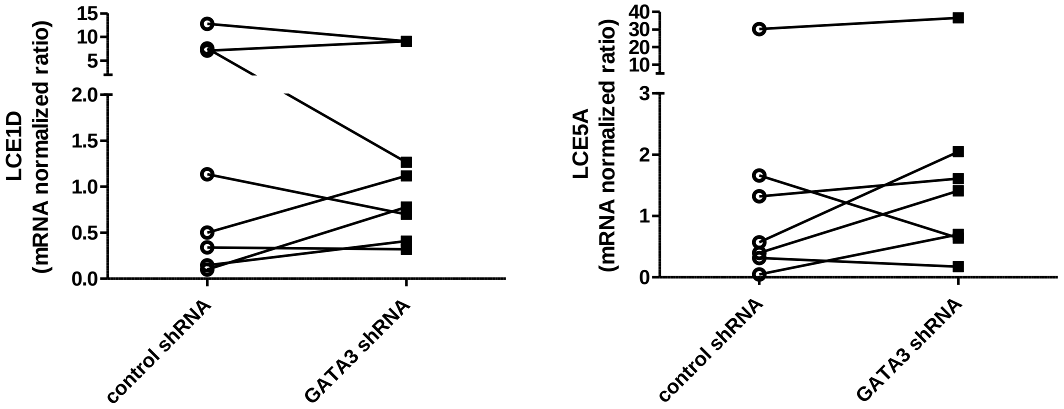

Supplementary Figure S2: LCE1D and LCE5A mRNA expression in GATA3 silenced keratinocytes. GATA3 silenced and control treated keratinocytes were analyzed by qRT-PCR for the expression of LCE1D and LCE5A. Relative mRNA expression levels normalized to PGK1 are shown. n=7-8 independent experiments with keratinocytes from different donors.
